# Argonaute 2 stabilization of microRNAs controls adult neurogenesis and oligodendrogenesis

**DOI:** 10.4103/NRR.NRR-D-24-00043

**Published:** 2025-11-25

**Authors:** Xianshuang Liu, Wanlong Pan, Michael Chopp, Baoyan Fan, Xinli Wang, Julie Landschoot-Ward, Min Wei, Qinge Lu, Helen Liu, William Golembieski, Sutapa Santra, Zheng Gang Zhang

**Affiliations:** 1 Department of Neurology, Henry Ford Hospital, Detroit, MI, USA; 2 Department of Physics, Oakland University, Rochester, MI, USA

**Keywords:** Argonaute 2, microRNA, neural stem cells, neurogenesis, oligodendrogenesis, stability, stroke, subventricular zone

## Abstract

MicroRNAs regulate neural stem cell function. Argonaute 2 protein, constituent of the RNA-induced silencing complex, plays an important role in regulating microRNA function for post-transcriptional gene silencing. Although Argonaute 2 and microRNAs are recognized as central regulators of RNA-induced silencing complex, their precise role in adult neural stem cell function has remained unclear. In particular, it was not known whether Argonaute 2 is required for sustaining neural stem cell proliferation, neurogenesis, and oligodendrogenesis in the adult brain, or how its loss might influence recovery after ischemic injury. The present study examined the effect of Argonaute 2 deletion in adult neural stem cells on neurogenesis and oligodendrogenesis. Adult transgenic mice with conditional and inducible ablation of Argonaute 2 in Ascl1-lineage neural stem cells exhibited the reduction of neurogenesis in the ventricular–subventricular zone of the lateral ventricle and in the subgranular zone of the dentate gyrus, as evidenced by a decrease in neural stem cell proliferation and neuroblast numbers. Argonaute 2 deletion also reduced oligodendrogenesis in the corpus callosum, as indicated by the reduction of oligodendrocyte progenitor cell proliferation and the number of mature oligodendrocytes. Additionally, deleting Argonaute 2 in adult neural stem cells of ischemic mice exacerbated impairments of sensorimotor and cognitive functions. Mechanistically, Argonaute 2 ablation in neural stem cells reduced the stability of mature microRNAs and downregulated genes involved in the Shh (Sonic Hedgehog), Notch, and TGFβ (transforming growth factor beta) signaling pathways, which regulate the functions of neural stem cells. Collectively, our study demonstrates that Argonaute 2 is essential for adult neural stem cell-mediated neurogenesis and oligodendrogenesis, with its deletion worsening recovery after ischemia. By revealing that Argonaute 2 stabilizes mature microRNAs, our work uncovers a novel mechanism of neural stem cell regulation and highlights Argonaute 2 as a potential therapeutic target for neurodegenerative and ischemic brain diseases.

## Graphical abstract

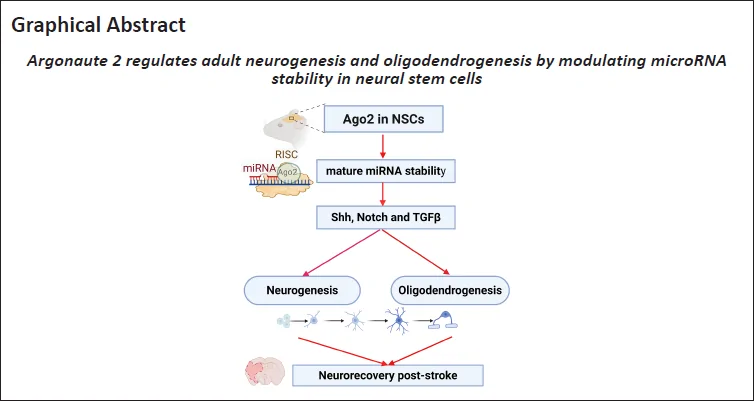

## Introduction

In the adult rodent brain, neurogenesis occurs primarily in the ventricular–subventricular zone (V-SVZ) of the lateral ventricle and in the subgranular zone (SGZ) of the dentate gyrus (Gage et al., 1995; Kirschenbaum et al., 1999; Hussain et al., 2024; Elliott et al., 2025). Neural stem cells (NSCs) in the V-SVZ also produce nonmyelinating oligodendrocyte progenitor cells (OPCs) that disperse throughout the corpus callosum (CC) and the neighboring striatum (Parras et al., 2004; Menn et al., 2006; Zhang et al., 2011). Stroke-induced neurogenesis and oligodendrogenesis are involved in ischemic brain repair processes and functional recovery (Zhang et al., 2001; Arvidsson et al., 2002; Nagase et al., 2023; Geng et al., 2026). For example, ablation of newly generated neuroblasts in ischemic brain reduces spontaneous functional recovery, whereas enhancement of endogenous oligodendrogenesis facilitates brain repair processes and improves neurological outcome in a mouse model of middle cerebral artery occlusion (MCAO) (Iwai et al., 2010; Jin et al., 2010; Wang et al., 2012; Geng et al., 2026). However, spontaneous neurogenesis and oligodendrogenesis in response to stroke are insufficient to restore neurological function (Arvidsson et al., 2002; Zhang et al., 2010). Elucidating cellular and molecular mechanisms underlying induced adult neurogenesis could potentially lead to the development of new therapies to amplify endogenous neurogenesis and oligodendrogenesis, consequently leading to the improvement of neurological function during stroke recovery.

MicroRNAs (miRNAs) are small non-coding RNAs, approximately 21 nucleotides in length, and have been implicated in numerous cellular processes (Shang et al., 2023). MiRNA precursors are processed into mature miRNAs by the RNAase III enzyme Dicer (Shang et al., 2023). The function of miRNAs is primarily restricted to the RNA-induced silencing complex (RISC), in which miRNAs bind to target mRNAs and inhibit their translation or elicit their degradation (Iwakawa and Tomari, 2022). Argonaute (Ago) proteins are core factors in RISCs. Ago 2 (EIF2C2) is a distinct member of the Ago family with catalytic activity and is essential for small RNA-mediated gene silencing (Meister, 2013; Nakanishi, 2022). Ago2 is associated with the development and human diseases including tumorigenesis, stress, angiogenesis, diabetes and hypoxia (Zhang et al., 2021; Liu et al., 2023; Powell et al., 2023; Kim et al., 2025; Wang et al., 2025). Mutations of Ago2 contribute to human cancer development by deregulating miRNAs. Mice deficient in Ago2 are embryonic lethal and exhibit defective neural tube and heart development (Boyer et al., 2005), suggesting that Ago2 plays an essential role in stem cells. The effect of Dicer, on neurogenesis has been well studied; however, whether Ago2 regulates adult neurogenesis and oligodendrogenesis in particular after stroke, remains unknown.

Stroke alters the profiles of miRNAs in the brain and in blood samples obtained from humans and rodents (Jeyaseelan et al., 2008; Liu et al., 2010; Mens et al., 2021; Todoran et al., 2023). NSCs and oligodendrocytes express miRNAs, which regulate neurogenesis and oligodendrogenesis (Zhao et al., 2010; Zolboot et al., 2021; Ngo and Kothary, 2022; Rashidi et al., 2023). We have demonstrated that stroke substantially changes the mature miRNA profiles in NSCs, and that miR-124a, miR-146, and the miR17-92 cluster regulate stroke-induced neurogenesis (Liu et al., 2011, 2013). Although stroke alters numerous miRNAs, the regulation of stroke-altered miRNAs remains poorly understood.

In the present study, using a transgenic mouse line, we provide the first direct evidence that Ago2 is required for adult NSC-driven neurogenesis and oligodendrogenesis, and that its loss worsens functional recovery after stroke. Our study identifies Ago2 as a potential therapeutic target to enhance brain repair in stroke and related neurodegenerative disorders.

## Methods

### Animals

All experimental procedures were done in accordance with the NIH Guide for the Care and Use of Laboratory Animals and approved by the Institutional Animal Care and Use Committee (IACUC# 00001455, approved on December 16, 2022) of Henry Ford Hospital.

We first generated an Ascl1 lineage NSC reporter line, *Ascl1-Cre*^*ERT2*^*;Tom*, by crossing *Ascl1-Cre*^ERT2^ (Stock No. 012882, Jackson Lab, Bar Harbor, ME, USA) and *Rosa-TdTomato* (*Tom*) reporter mice (Jackson Lab, Stock No. 007914). To generate conditional and inducible *Ago2* knockout mice, *Ascl1-Cre*^*ERT2*^*;Tom* reporter mice were bred with *Ago2*^*flox/flox*^ (*Ago2*^f/f^) mice (Jackson Lab, Stock No. 016520) to produce *Ascl1-Cre*^*ERT2*^*;Ago2*^*flox/flox*^*;Tom* mice. The genotype of all transgenic mice used in the present study was confirmed prior to experiments. Transgenic mice at age of 2–3 months were treated with tamoxifen (100 mg/kg/d, intraperitoneally) for 5 consecutive days to generate Ago2 reporter NSC mice and Ago2 knockout NSC mice. Two days after the last injection, these mice were subjected to middle cerebral artery occlusion (MCAO) and were euthanized 28 days post-MCAO. Mice and rats were maintained in polycarbonate type II cages within an individually ventilated and specific-pathogen–free animal facility, under controlled environmental conditions (temperature 20–24°C; relative humidity 40%–65%) and a 12-hour light/dark cycle. Standard chow and water were provided ad libitum.

### Middle cerebral artery occlusion

Adult specific pathogen-free male C57B/6J mice (2–3 months old, 20–30 g, Jackson Lab) were employed in this study. Only male mice were used in this study to minimize variability associated with hormonal fluctuations during the estrous cycle, which can influence behavioral, metabolic, and neurological outcomes (Beery and Zucker, 2011). Permanent right MCAO was induced. The surgical procedures were performed under full anesthesia. Mice were anesthetized with 3.5% isoflurane (Sigma-Aldrich, St. Louis, MO, USA) in a mixture of N_2_O:O_2_ (2:1) and maintained at 0.5%–1.5% isoflurane (Sigma-Aldrich) using a facemask. Briefly, under anesthesia, the right common carotid artery, external carotid artery, and internal carotid artery were exposed. An 8-0 nylon filament with an expanded silicone tip was gently advanced from the external carotid artery into the lumen of the internal carotid artery to block the origin of the middle cerebral artery (Liu et al., 2013).

### Tamoxifen administration

The *Ascl1-Cre*^ERT2^*;Ago2*^flox/flox^*;Tom* mice were intraperitoneally injected with tamoxifen (100 mg/kg/d, Sigma-Aldrich) for 5 consecutive days. Two days after the last injection, these mice were subjected to MCAO, and they were euthanized 28 days post-MCAO.

### 5-Bromo-deoxyuridine labeling

To examine the fate of NSCs, we employed a 5-bromo-deoxyuridine (BrdU; chasing approach (Wojtowicz and Kee, 2006; Zhang et al., 2014b). Briefly, animals were intraperitoneally injected with BrdU (100 mg/kg, Sigma-Aldrich) daily for 5 consecutive days starting 2 days after the final tamoxifen injection. BrdU is the thymidine analog that is incorporated into the DNA of dividing cells during the S phase, as previously described (Zhang et al., 2011). Animals were sacrificed 28 days after the initial BrdU injection.

### Culture of primary neural stem cells

NSCs from the ischemic side of V-SVZ were dissociated from adult C57B/6J mice, as previously described (Reynolds and Weiss, 1992; Liu et al., 2011). The cells were plated at a density of 2 × 10^4^ cells/mL in growth medium. The growth medium contained DMEM/F-12 medium (Thermo Fisher Scientific, Waltham, MA, USA), 20 ng/mL of epidermal growth factor (R&D System, Minneapolis, MN, USA), and basic fibroblast growth factor (R&D System). The generated neurospheres (primary spheres) were passaged by mechanical dissociation. V-SVZ cells from ischemic mice were extracted 7 days after MCAO, a peak time of increase of neurogenesis (Marlier et al., 2015). Given that the original V-SVZ cells at passage 1 may contain other cells and tissue debris, V-SVZ cells used in all experiments were from passages 2–5, and the passage time was approximately 5 days, based on the sizes of NSC neurospheres.

### Primary oligodendrocyte progenitor cell culture

For *in vitro* experiments, primary OPCs were isolated from embryonic day-18 Wistar rats (Charles River, Wilmington, MA, USA), as previously described (O’Meara et al., 2011). Briefly, embryonic Wistar rats were decapitated with intraperitoneal injection of ketamine (160 mg/kg) and xylazine (26 mg/kg). Cortices were dissected and then rinsed and incubated at 37°C for 15 minutes with 0.01% trypsin and DNase (Thermo Fisher Scientific). The tissue was mechanically dissociated and passed through a 40-μm sterile cell strainer to remove insoluble debris. Cells were seeded in poly-D-lysine–coated culture flasks (Sigma-Aldrich) and maintained in DMEM supplemented with 20% fetal bovine serum until confluence was reached (approximately 10 days). At this stage, a confluent astrocytic monolayer developed, with a layer of OPCs on top. To remove dead cells and microglia, cultures were shaken at 9 × *g* for 1 hour, followed by overnight shaking at 9 × *g* for 16 hours to detach OPCs. The isolated OPCs were then replated onto poly-D,L-ornithine–coated dishes in serum-free DMEM containing human Apo-transferrin (Sigma-Aldrich), sodium selenite, D-biotin, L-glutamine, penicillin-streptomycin, hydrocortisone, BSA, and insulin. Proliferation medium was further supplemented with OPC mitogens, including PDGF-AA and NT-3 (PeproTech, Cranbury, NJ, USA), whereas differentiation medium was prepared with triiodothyronine (T3; Sigma-Aldrich) and ciliary neurotrophic factor (CNTF, 10 ng/mL; PeproTech) in the absence of OPC mitogens.

### Neurosphere assay

To examine the effect of Ago2 knockdown on V-SVZ cell proliferation, single cells at a density of 10 cells/µL were incubated in the growth medium for 7 days and BrdU (30 μg/mL, Sigma-Aldrich) was added 18 hours before the termination of incubation. The percentage of BrdU-positive cells was measured.

To examine the effect of Ago2 knockdown on V-SVZ cell differentiation, neurospheres were plated directly onto laminin-coated glass coverslips in the growth medium described above, in which epidermal growth factor and basic fibroblast growth factor were replaced with 2% fetal bovine serum, hereafter referred to as the differentiation medium. Every 4 days, one-half of the medium was replaced with fresh medium. Incubation was terminated 10 days after plating, and immunostaining was performed to detect neuronal and oligodendrocyte phenotypes to evaluate cell differentiation.

### Transfection of DNA and RNA by electroporation

RNA oligonucleotides or DNAs (Dharmacon, Chicago, IL, USA) were transfected into NSCs by electroporation. Scrambled siRNA (Dharmacon) was employed as a negative control for siRNA transfection. Briefly, using a Nucleofector^TM^ kit which included the Nucleofector® solutions and supplements (Lonza, Basel, Switzerland), RNA oligonucleotides (200 pmol) or DNAs were mixed with 100 μL of nucleofector solution and cell-RNA mixtures were transferred into a cuvette and electroporated using program A33 for NSC transfection.

### Quantification of mRNA by quantitative reverse transcription-polymerase chain reaction

RNAs extracted from the V-SVZ were reverse transcribed using M-MLV reverse transcriptase (Thermo Fisher Scientific). For each sample, 2 μg of RNA was reverse transcribed at 42°C for 30 minutes in a reaction containing 1 μg oligo(dT) or gene-specific primers, 5× first-strand buffer, 100 mM DTT, 10 mM dNTPs, RNase inhibitor (Thermo Fisher Scientific), and M-MLV enzyme. The resulting cDNAs were evaluated for optimal dilution prior to quantitative reverse transcription-polymerase chain reaction (qRT-PCR). qRT-PCR reactions (20 μL) contained diluted cDNA, SYBR Green qPCR Master Mix (Thermo Fisher Scientific), and 10 μM primers. Amplification was performed on an ABI 7000 system (Thermo Fisher Scientific) with the following cycling conditions: 95°C for 4 minutes, followed by 40 cycles consisting of a 20°C/s temperature transition to 95°C (30 seconds), 62°C (45 seconds), and subsequent melting curve analysis. All reactions were run in triplicate with β-actin as the internal reference gene. Median Ct values were used for analysis, and relative expression levels were calculated using the 2^–ΔΔCt^ method.

### RNA sequencing

Total RNAs isolated from the V-SVZ tissues were quantified using a NanoDrop ND-1000 instrument (Thermo Fisher Scientific). Total RNA (1–2 μg) was used to prepare the sequencing library in the following steps: 1) Total RNA is enriched by oligo(dT) magnetic beads (rRNA removed); 2) RNA sequencing (RNA-seq) library preparation using KAPA Stranded RNA-Seq Library Prep Kit (Illumina, San Diego, CA, USA), which incorporates dUTP into the second cDNA strand and renders the RNA-seq library strand-specific. The completed libraries were qualified with Agilent 2100 Bioanalyzer (Agilent, Santa Clara, CA, USA) and quantified by absolute quantification qPCR method. To sequence the libraries on the Illumina HiSeq 4000 instrument, the barcoded libraries were mixed, denatured to single stranded DNA in NaOH, captured on Illumina flow cell, amplified *in situ*, and subsequently sequenced for 150 cycles for both ends on Illumina HiSeq instrument. Image analysis and base calling were performed using Solexa pipeline v1.8 (Off-Line Base Caller software, v1.8, Illumina). The trimmed reads were aligned to reference genome using Hisat2 software (https://daehwankimlab.github.io/hisat2/). The transcript abundances for each sample were estimated with StringTie (https://github.com/gpertea/stringtie) and the fragments per kilobase of transcript per million mapped reads (FPKM) value for gene and transcript level were calculated with R package Ballgown (https://github.com/alyssafrazee/ballgown). The differentially expressed genes and transcripts were filtered using R package Ballgown (Frazee et al., 2015). The novel genes and transcripts were predicted from assembled results by comparing to the reference annotation using StringTie and Ballgown. Correlation analyses were based on gene expression level, Hierarchical Clustering, Gene Ontology, Pathway analysis, scatter plots were performed with the differentially expressed genes in R (https://www.r-project.org/) or Python (https://www.python.org/) for statistical computing and graphics.

### Polymerase chain reaction array

The Mouse Stem Cell PCR Array (Qiagen, Germantown, MD, USA) was used to profile the expression of 84 stem cell–related genes. Experiments were performed according to the manufacturer’s instructions. mRNA expression levels were normalized to the housekeeping genes included in the array, which served as endogenous controls. Fold-regulation values for each transcript were calculated by inputting the threshold cycle (Ct) values into the manufacturer’s web-based software. Fold-regulation values below 1 indicated negative fold change (downregulation).

### Quantification of mature miRNAs by quantitative reverse transcription-polymerase chain reaction

Individual reverse transcription and TaqMan® miRNA assays were performed to test miRNA expression in NSCs on an Applied Biosystems 7000 Instrument (Thermo Fisher Scientific). Mature miRNA sequences for TaqMan primers are listed in **Table 1**. Reverse transcription reactions (15 μL) were prepared using 1–10 ng of total RNA isolated with TRIzol (Qiagen), 5 U of MultiScribe Reverse Transcriptase, 0.5 mM each dNTP, 1× Reverse Transcription buffer, 4 U RNase Inhibitor, and nuclease-free water. The reactions were incubated at 16°C for 30 minutes, 42°C for 30 minutes, and 85°C for 5 minutes, and then stored at 4°C until use in TaqMan assays. TaqMan real-time PCR reactions (20 μL) contained 1× TaqMan Universal PCR Master Mix (No AmpErase UNG), 1× TaqMan miRNA assay, 1.33 μL undiluted cDNA, and nuclease-free water. Each assay was performed in triplicate for each sample. Relative expression levels were calculated using the 2^–ΔΔCt^ method, with U6 snRNA (Applied Biosystems) as the endogenous control and wild-type (WT) samples as the calibrator. Three independent experiments were conducted. PCR amplification was performed on the Standard 7000 system using the default cycling protocol, excluding the 50°C incubation step. The reactions were incubated at 95°C for 10 minutes, followed by 40 cycles of 95°C for 15 seconds and 60°C for 1 minute, with fluorescence readings collected during the 60°C step.

### Western blotting

Samples isolated from NSCs or brain tissues from Ago2 knockout or male C57B/6J mice were lysed with RIPA buffer supplemented with 1% proteinase and centrifuged at 12,000 × *g* for 15 minutes to remove cell debris. Protein concentration in supernatant was determined using a Bicinchoninic acid (BCA) assay (Thermo Fisher Scientific). Total protein (30 μg) from each sample was separated by SDS-PAGE and transferred to nitrocellulose membranes. After blocked with 3% BSA blocking buffer, the membranes were incubated overnight at 4°C with the following primary antibodies: mouse anti-β-actin (1:5000, Abcam, Waltham, MA, USA, Cat# ab6276, RRID: AB_2223210), rabbit anti-Ago1 (1:1000, Cell Signaling Technology, Danvers, MA, USA, Cat# 5053S), rabbit anti-Ago2 (1:500, Abcam, Cat# ab32381, RRID: AB_867543), rabbit anti-Ago3 (1:500, Sigma, Cat# SAB4200112, RRID: AB_10634602), rabbit anti-Ago 4 (1:500, Cell Signaling Technology, Cat# 6913S), rabbit red fluorescent protein (anti-RFP, 1:500, Rockland, Limerick, PA, USA, Cat# 600-401-379, RRID: AB_2209751), rabbit anti-Cre (1:500, Abcam, Cat# ab216262), rabbit anti-myelin proteolipid protein (mPLP, 1:500, Abcam, Cat# ab28486, RRID: AB_776593), rat anti-myelin basic protein (MBP; 1:500, Chemicon, Limerick, PA, USA, Cat# MAB386, RRID: AB_94975). After incubation with secondary antibodies, the signals were visualized by an enhanced ECL substrate (Thermo Fisher Scientific). Band intensity in Western blot images was quantified with ImageJ software (Version 1.54p, https://imagej.net/ij/).

### MiRNA decay assay

MiRNA stability was examined in NSCs treated with Actinomycin D (AMD; 10 μg/mL), a transcriptional inhibitor, in comparison with cells treated with the DMSO vehicle control. Additionally, miRNA expression levels were analyzed in NSCs following Ago2 knockdown or overexpression.

### Bioinformatics analysis

To assay the gene targets of differentially expressed miRNAs, we used three of the leading miRNA target prediction algorithms miRanda (http://microrna.sanger.ac.uk/sequences/), PicTar (http://pictar.mdc-berlin.de/), TargetScan (http://www.targetscan.org/). To perform an enrichment analysis of predicted target genes of miRNAs in biological pathways, Ingenuity Pathway Analysis (IPA) algorithm, a web-based application, was used. For peak finding and motif analysis, we first trimmed data to remove adapters and discard low-quality reads, keeping reads that are 15 bp or longer. Next, we aligned the reads to rat genome using Bowtie alignment program, and mapped reads to genes and to 3′UTR.

### Immunocytochemistry and quantification

For cell proliferation assay, the neurospheres were dissociated into the single NSCs and seeded on 1% laminin (Thermo Fisher Scientific) -coated 8-well chambered cell culture slides (Thermo Fisher Scientific) at a density of 105 cells per well in proliferation medium. BrdU (5 μM, Sigma-Aldrich) was added into medium and cultured for 18 hours. Cells were then washed and fixed in 4% paraformaldehyde for 10 minutes, incubated with 50% formamide for 30 minutes at 65°C, washed with 1 M HCl for 10 minutes at 37°C, and incubated in 0.1 M borate buffer for 3 minutes. Cells were stained with rabbit anti-BrdU (1:2000, Abcam, Cat# ab152095, RRID: AB_2813902) overnight at 4°C and then followed Cy3-conjugated secondary antibodies. Nuclei were stained by 4′,6′-diamidino-2-phenylindole (DAPI; 1:5000, Abcam, Cat# ab228549).

To analyze NSC differentiation, cells were cultured in differentiation medium consisting of growth medium supplemented with 2% fetal bovine serum for 5 days, then fixed, incubated with blocking buffer, and stained with mouse anti–neuron-specific class III β-tubulin (Tuj1; 1:300, Abcam, Cat# ab18207, RRID: AB_444319), mouse anti-O4 (1:800, R&D System, Cat# MAB1326, RRID: AB_357617), or mouse anti-BrdU (1:100, Dako, Santa Clara, CA, USA, Cat# M0744) antibody overnight at 4°C. The next day, cells were washed and incubated with the secondary antibodies and DAPI, as described above. Images were taken in 10 view fields/well and 3 wells/group. The percentage of positive cells was quantified.

### Brain tissue preparation and single immunohistochemistry

Mice and rats were perfused with saline and followed by perfusion with 4% paraformaldehyde. The brains were removed and fixed further in 4% formaldehyde for 4 hours at 4°C, and then transferred into 30% sucrose in phosphate-buffered saline for 24 hours. The brains were embedded and frozen in optimal cutting temperature compound. A series of 4-μm-thick brain coronal sections were cut in a cryostat (Thermo Electron Corporation, Waltham, MA, USA) from the bregma (1.18 mm to –0.82 mm) for the mice.

After fixation with formaldehyde and endogenous peroxidase activity quenching, sections were incubated with bovine serum albumin for 1 hour. Then, they were incubated with primary antibody for mouse anti-BrdU (1:200, Dako, Cat# M0744, RRID: AB_10013660) at 4°C overnight. The next day, the sections were treated with mouse monoclonal biotinylated secondary antibody (1:200; Vector, Burlingame, CA, USA, Cat# BA-9200-1.5) for 2 hours at room temperature. Immunoreactivities were developed in horseradish peroxidase-streptavidin-biotin complex solution (Vectastain ABC kit; Vector) for 30min and then incubated with 3,3′-diaminobenzidine (DAB; Vector).

### Double-fluorescence immunohistochemistry

Every fifth section was used for immunohistochemistry, as previously described (Zhang et al., 2011). Briefly, the sections were incubated with the following antibodies: mouse anti-BrdU (1:100, Dako, Cat# M0744, RRID: AB_10013660), mouse anti-nestin (1:100, BD Bioscience, Franklin, NJ, USA, Cat# BDB561551), mouse anti-RB1 inducible coiled-coil 1 (CC1; also termed as APC, 1:200, Calbiochem, San Diego, CA, USA, Cat# OP44, RRID: AB_2242783), rabbit anti-neural/glial antigen 2 (NG2; 1:200, Millipore, Burlington, MA, USA, Cat# AB5320, RRID: AB_91789), mouse anti-doublecortin (DCX; 1:200, Santa Cruz Biotechnology, Santa Cruz, CA, USA, Cat# SC-271390, RRID: AB_10610966), goat anti-SRY-box transcription factor 2 (SOX2; 1:500, Santa Cruz Biotechnology, Cat# SC-17320, RRID: AB_2286684), or mouse anti-O4 (1:800, R&D System, Cat# MAB1326, RRID: AB_357617) at 4°C overnight and then with an anti-goat/rabbit/mouse IgG FITC- or Cy3-conjugated secondary antibody (1:200, Jackson ImmunoResearch, West Grove, PA, USA) for 2 hours at room temperature. Cell nuclei were stained with DAPI. Double immunofluorescent images were acquired using a Zeiss (Zeiss, Thornwood, NY, USA) LSM 510 Meta-NLO system. To evaluate the results of neurogenesis and oligodendrogenesis, the positively stained cells were counted in the V-SVZ, dentate gyrus, and CC in five coronal sections per brain. The staining intensity for Ago2 DAB staining was measured.

### Learning and memory tests

The Novel object recognition test has been widely used to investigate spatial and nonspatial memory as well as temporal order memory (Lueptow, 2017). The test consisted of three phases:

1) Pre-habituation procedures. Mice were removed from group-housing cages and rehoused singly in identical cages containing sawdust bedding and removable wire tops. Once singly housed, the mice remained in these cages for the duration of the experiment. During the initial 24-hour familiarization period, four wooden beads (20 mm in diameter, each with a central hole) were introduced into the test cages. These beads acquired the individual animal’s odor and served as familiar odor stimuli in subsequent testing. Housing the animals with the beads during this period allowed for familiarization with both the test environment and the beads themselves. Additional beads were placed in the cages of three designated odor-donor groups (3 mice/cage), which were not cleaned for 1 week to allow accumulation of cage-specific novel odors. Donor cages were counterbalanced such that each odor source could serve as either a recent novel odor (N1) or a new novel odor (N2) across experimental subjects.

2) Habituation to the first novel odor. Following the 24-hour familiarization period, the four familiar odor beads were removed for 1 hour. After this interval, three familiar beads (collected from the animal’s home cage 1 hour earlier) and one novel odor bead (N1) obtained from a donor cage were introduced into the test cage. Mice were exposed to these four beads across three 1-minute trials, separated by 1-minute intervals during which the beads were removed. For each trial, the three familiar beads and the N1 bead were placed in the center of the test cage. The trial began upon the animal’s first approach to a bead and lasted 1 minute. Exploration time for each bead was recorded.

3) Odor-recognition memory assessment. Twenty-four hours after the habituation phase, odor recognition was tested. Mice were presented with four beads: one previously encountered novel odor bead (N1), one new novel odor bead (N2) from a different donor cage, and two familiar beads from their own cage. The procedure followed was identical to that described in the habituation phase. The N2 ratio was calculated as time spent exploring the novel wood bead (N2) relative to the total time spent exploring all objects.

Morris water maze (MWM) test: To assess spatial learning performance, we employed the MWM test, as previously described (Fan et al., 2018). The MWM test was carried out during the last 5 days before the mice were killed, and automated data were recorded and analyzed using the HVS Image 2020 Plus Tracking System (US HVS Image).

Learning and memory tests as outlined above were performed at 28 days after the final tamoxifen injection.

### Neurological outcome assay

Adhesive removal test: Mice were tested for somatosensory deficit with the adhesive removal test, as previously described (Zhang et al., 2014a). Briefly, 2 pieces of adhesive-backed round paper dots (113 mm^2^) were used as bilateral tactile stimuli, which were adhered on each forelimb at the distal radial region of the wrist. The mean time (seconds) for mice to remove the stimulus from the left forelimb was recorded based on three trials per testing day.

Foot-fault test: Mice were tested for forelimb placement dysfunction with the Foot-fault test, as previously described (Zhang et al., 2014a). Briefly, animals were placed on an elevated grid surface (85.5 × 2.5 cm^2^), with grids of different sizes and shapes. While moving along the grid, slips and falls of animals’ paws through the wire grid were counted as foot-faults. The left forelimb foot-faults and the total steps made by both forelimbs were simultaneously recorded. Data are presented as a percentage of left forelimb foot-faults relative to the total steps.

Modified neurological severity score (mNSS) test: Mice were tested for motor (muscle status, abnormal movement), sensory (visual, tactile, and proprioceptive), reflex, and balance dysfunctions with the mNSS, as previously described (Zhang et al., 2014a). Neurological deficit was rated on a scale of 18 (0 for normal, 18 for maximal deficit). In the severity scores of injury, one point is awarded for each abnormal behavior or for lack of a tested reflex; thus, the higher the score, the more severe the injury.

Neurological functions were measured at 1, 3, 7, 14, and 28 days post-MCAO.

### Statistical analysis

Data are presented as mean ± standard error of the mean (SEM). GraphPad Prism software (GraphPad Software, San Diego, CA, USA) was used for statistical analyses. Statistical significance was determined using two-tailed Student’s *t*-tests for two-group comparisons, and two-way analysis of variance with repeated measures was performed for multiple group comparisons. A value of *P* < 0.05 was taken as significant. Quantification was performed from at least three independent experiments.

## Results

### Conditional ablation of Argonaute 2 in Ascl1-lineage neural stem cells reduces adult neurogenesis and oligodendrogenesis

**Figure 1:**
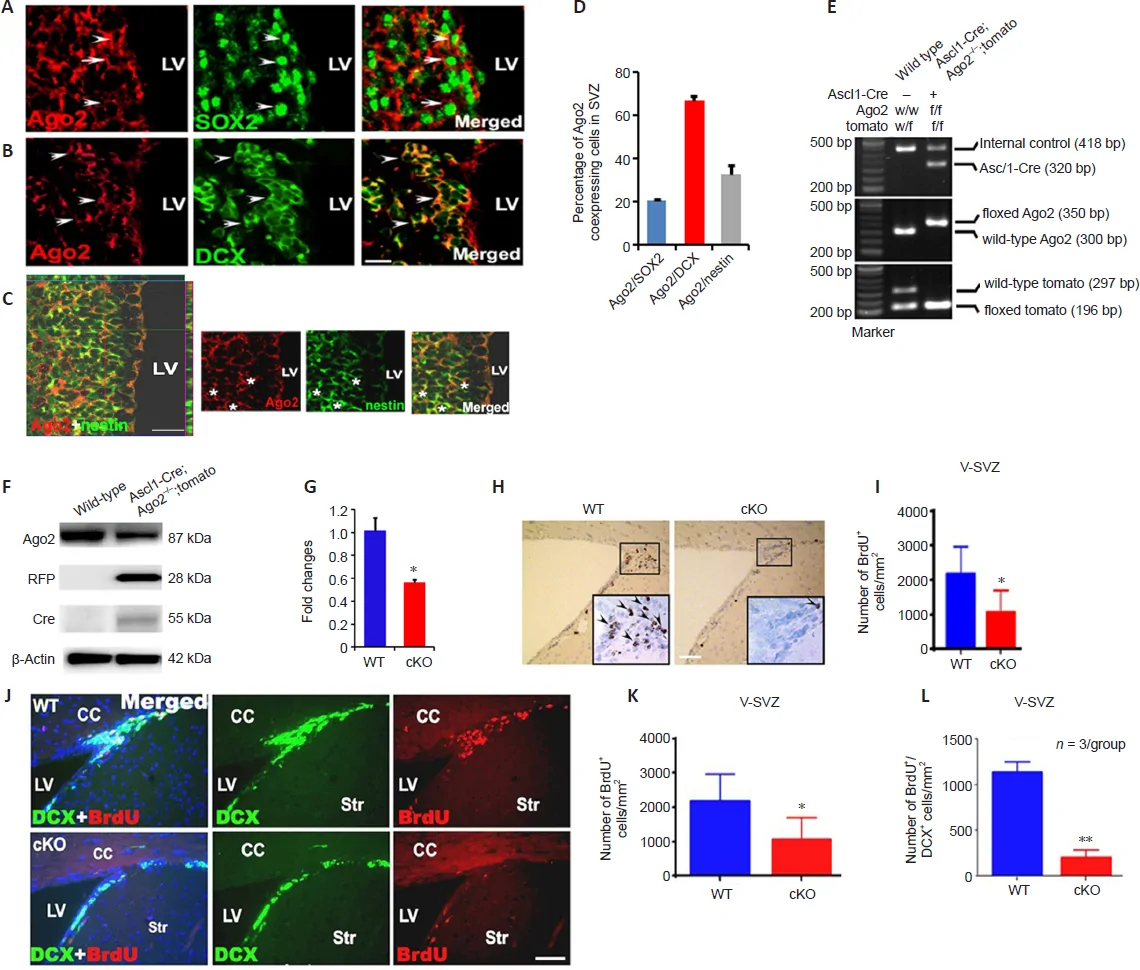
Ablation of Ago2 in neural stem cells reduces neurogenesis in the V-SVZ. (A–C) Double immunofluorescent staining on coronal brain sections shows that Ago2^+^ cells in the SVZ are SOX2 (A, arrows), DCX (B, arrows), and nestin (C, asterisk) positive. Scale bars: 20 μm (A–C). (D) The percentage of Ago2 coexpressed with SOX2, DCX, and nestin positive cells in the V-SVZ. (E) PCR products of tail DNA identifies the presence of Cre together with either Ago2 or Tomato (Tom) or both, wild-type (WT) and Ago2 conditional knockout (cKO, *Ascl1-Cre;Ago2*^*–/–*^*;Tom*) mouse lines. Marker: 50 bp DNA ladder marker (NEB). w/f: wild type/floxed. (F, G) Western blot analysis shows Ago2, Cre, RFP protein levels (F) and quantification of Ago2 (G) in V-SVZ tissues isolated from *Ago*^*+/+*^*;Tom* and *Ascl1-Cre;Ago2*^*–/–*^*;Tom* transgenic mice. (H–J) Ago2 cKO mice displayed a significantly reduced proliferating BrdU^+^ (H, I) and differentiated BrdU^+^/DCX^+^ neuroblasts (J) in the V-SVZ compared with WT mice. This analysis was conducted 28 days after the final tamoxifen injection. Scale bars: 20 μm (H and J). (K, L) Quantification of BrdU^+^ (K) and BrdU^+^/DCX^+^ cells (L) in Ago2 cKO mice and WT mice. *n* = 3/group. Data are presented as mean ± SEM. *P < 0.05, **P < 0.01, *vs*. WT. Ago2: Argonaute 2; BrdU: bromodeoxyuridine; CC: corpus callosum; cKO: conditional knockout; DCX: doublecortin; LV: lateral ventricle; RFP: red fluorescent protein; SOX2: SRY-box transcription factor 2; Tom: Tomato; V-SVZ: ventricular–subventricular zone; WT: wild-type.

Adult NSCs mainly reside in the V-SVZ of the lateral ventricle and in the SGZ of the dentate gyrus of the rodent (Gage et al., 1995; Kirschenbaum et al., 1999; Hussain et al., 2024; Elliott et al., 2025). Double immunofluorescent staining showed that SOX2^+^ NSCs (Pagin et al., 2021) or nestin^+^ NSCs (Tong and Yin, 2024) in the V-SVZ were Ago2^+^ cells (**Figure 1A** and **D**), while DCX^+^ neuroblasts (Porter et al., 2022) were also Ago2^+^ (**Figure 1B** and **D**), indicating that adult NSCs and neuroblasts express Ago2. Using adult *Ascl1-Cre*^*ERT2*^*;Ago2*^*f/f*^*;Tom* mice, we assessed the Ago2 function in NSCs by inducible and conditional ablation of Ago2 in Ascl1 lineage NSCs with tamoxifen (**Figure 1E**). Western blot analysis of V-SVZ tissues 5 days after tamoxifen showed that Ago2 protein levels were markedly reduced, while RFP was induced in *Ascl1-Cre*^*ERT2*^*;Ago2* ^*f/f*^*;Tom* mice compared to those in WT litters (**Figure 1F** and **G**), indicating the ablation of Ago2. Thereafter, we termed the transgenic mice as Ago2 conditional knockout (cKO) mice.

**Figure 2:**
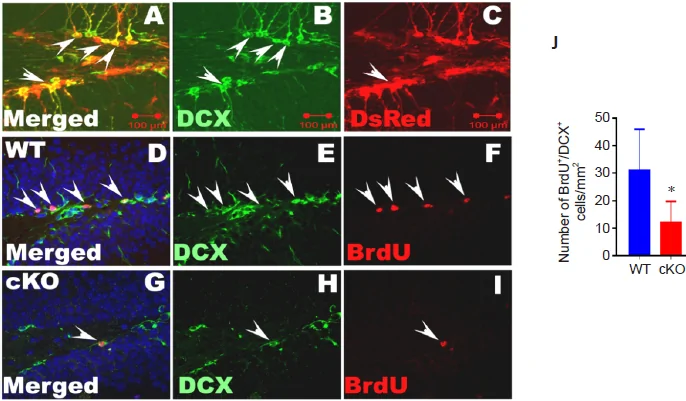
Conditional deletion of Ago2 in neural stem cells reduces hippocampal neurogenesis. (A–C) Ascl1-lineaged cells as indicated by DsRed^+^ were co-immunoreactive with DCX^+^ neuroblasts. (D–I) Compared with WT mice (D–F), Ago2 cKO mice (G–I) exhibited a marked reduction of proliferating BrdU^+^ and differentiated DCX^+^ neuroblasts in the subgranular zone of the dentate gyrus. Scale bars: 100 μm. (J) Quantification of BrdU^+^/DCX^+^ neuroblasts in Ago cKO mice and WT mice. *n* = 3/group. Data are presented as mean ± SEM. *P < 0.05, *vs*. WT. Ago2: Argonaute 2; BrdU: bromodeoxyuridine; cKO: conditional knockout; DCX: doublecortin; WT: wild-type.

To examine whether Ago2 regulated the NSC function, we used a BrdU chasing approach to determine NSC fate (Wojtowicz and Kee, 2006), in which BrdU was injected daily into the adult Ago2 cKO and WT mice for 5 days and the mice were sacrificed 28 days after the initial injection of BrdU. Double immunofluorescent analysis showed that compared to WT mice, Ago2 cKO mice dramatically reduced cell proliferation measured by the number of BrdU^+^ cells in the V-SVZ (**Figure 1H** and **I**). Also, we found that the number of BrdU^+^/DCX^+^ neuroblasts was decreased in the V-SVZ from Ago2 cKO mice (**Figure 1J–L**), suggesting that deletion of Ago2 reduced differentiation of BrdU-labeling NSCs into immature DCX^+^ neurons. Moreover, Ascl1 lineage cells labeled as DsRed^+^ were co-immunoreactive with DCX^+^ neuroblasts in the DG of the hippocampus (**Figure 2A–C**). The number of BrdU^+^/DCX^+^ neuroblasts in the DG was significantly reduced in Ago2KO mice compared to WT mice (**Figure 2D–J**). These data suggest that the ablation of Ago2 impairs adult neurogenesis.

**Figure 3:**
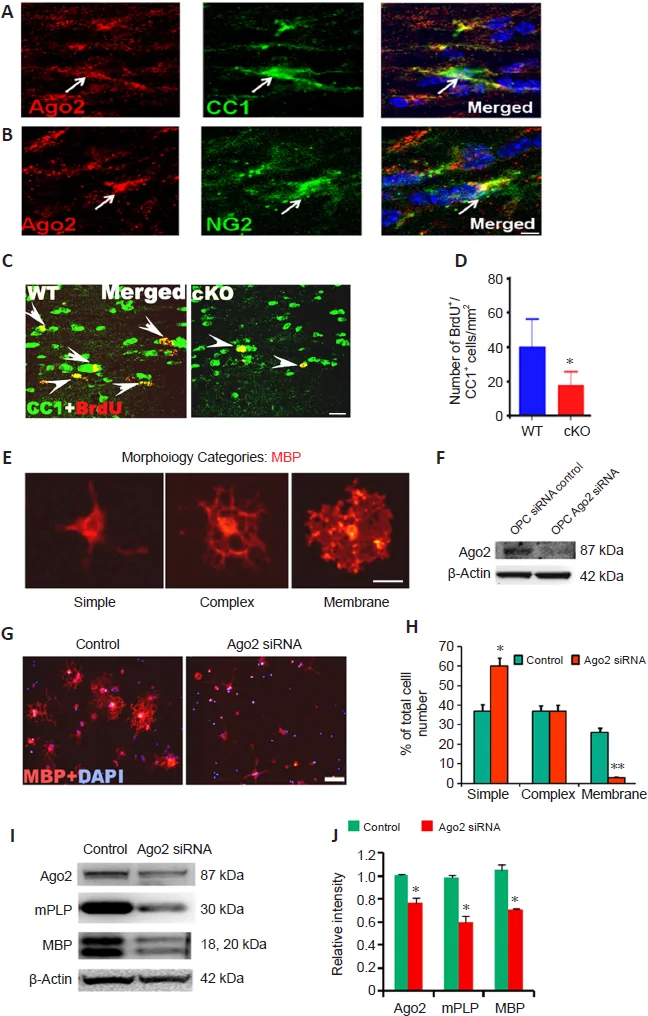
Effects of Ago2 ablation on OPCs. (A, B) Immunostaining on coronal brain sections shows that Ago2^+^ cells in the corpus callosum were CC1 (A, arrows) and NG2 (B, arrows) positive. (C) Immuno-staining shows the BrdU^+^/CC1^+^ cells in the corpus callosum from WT and Ago2 cKO. (D) Quantitative data of BrdU^+^/CC1^+^ cells in the corpus callosum. *n* = 3/group. (E) Extent of differentiation of OPCs was assessed by calculating the proportions of three levels of morphological complexity of MBP-expressing cells. Scale bar: 20 μm (A–C, E). (F) Ago2 was knocked down in the primary cultured OPCs transfected with siRNA-Ago2 or control siRNA. (G, H) The percentage of MBP^+^ cells with morphological change for the maturation state of differentiating oligodendrocytes from primary OPCs in control siRNA and siRNA-Ago2 groups. Scale bar: 100 μm (G). (I, J) Western blot shows that attenuation of Ago2 by siRNA suppressed myelin-related proteins. Data are presented as mean ± SEM. *P < 0.05, **P < 0.01, *vs.* WT or control. Ago2: Argonaute 2; BrdU: bromodeoxyuridine; CC1: RB1 inducible coiled-coil 1; cKO: conditional knockout; DAPI: 4′,6′-diamidino-2-phenylindole; LV: lateral ventricle; MBP: myelin basic protein; mPLP: myelin proteolipid protein; OPC: oligodendrocyte progenitor cell; siRNA: small interfering RNA; WT: wild-type.

Our previous study showed that Ascl1 lineage cells were detected not only in the V-SVZ, but also in the CC (Zhang et al., 2011). Our data showed that Ago2 colocalized with CC1^+^ cells (a marker of mature oligodendrocytes) and NG2^+^ cells (a marker of OPCs) in the CC (**Figure 3A** and **B**). We thus examined the effect of Ago2 ablation in Ascl1-lineage cells on adult oligodendrogenesis. Compared to WT mice, Ago2 cKO mice exhibited dramatically reduced cell proliferation measured by the number of BrdU^+^ cells and the number of BrdU^+^/CC1^+^ cells in the CC (**Figure 3C** and **D**).

To further verify the role of Ago2 in OPC differentiation and myelination, we employed primary cultured rat OPCs *in vitro*, and quantified the process complexity by analysis of MBP^+^ oligodendrocytes (**Figure 3E**). The knockdown of Ago2 was validated in rat OPCs transfected with Ago2 siRNAs compared to scrambled siRNAs (**Figure 3F**). Quantitative analysis detected a substantial decrease in the number of MBP^+^ cells with membrane sheets in Ago2 siRNA transfected oligodendrocytes compared to control siRNA-transfected cells, indicating decreased process branching and extensions from the cell body (**Figure 3G** and **H**). Western blot showed the myelin proteins including MBP and mPLP were decreased in Ago2 knockdown OPCs (**Figure 3I** and **J**). These data suggest that ablation of Ago2 in Ascl1 lineage cells inhibits the differentiation of OPCs into oligodendrocytes.

### Ablation of Argonaute 2 causes impaired learning and memory under non-ischemic conditions

**Figure 4:**
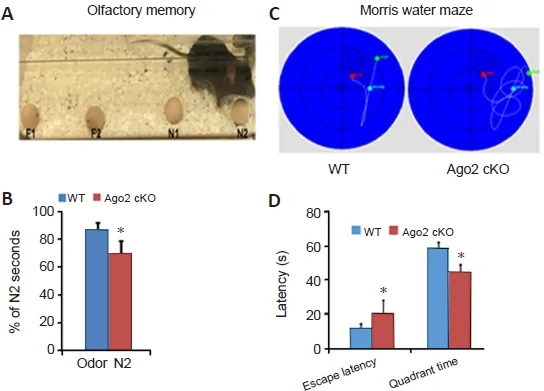
Effect of Ago2 cKO in Ascl1 lineage neural stem cells on cognition. (A) Schematic diagrams of wooden beads for odor-based novelty recognition test and circles indicate the wooden beads in the cage. (B) WT mice spent a lot of time exploring a novel bead (N2 bead). Compared to WT mice, Ago2 cKO mice spent significantly less time in exploring a new bead at 28 days after the final tamoxifen injection. Data are presented as the percentage of time spent on N2 relative to the total amount of time spent with all beads. N1 = familiar odor bead; N2 = unfamiliar novel odor bead. The Morris water maze was used to test spatial learning and memory. (C) Representative swimming paths on day 5 of the place navigation trial were recorded with a video tracking system. (D) Ago2 cKO mice spent significantly increased time to locate the hidden platform in the correct quadrant, as indicated by quantitative latency compared to WT mice. Ago2 cKO mice also spent much less time in the correct quadrant. *n* = 9 mice/group. Data are presented as mean ± SEM. *P < 0.05, **P < 0.01, *vs*. WT. Ago2: Argonaute 2; cKO: conditional knockout; WT: wild-type.

Under physiological conditions, NSCs in the V-SVZ travel along the rostral migratory stream to the olfactory bulb where they differentiate into mature interneurons and give rise to young oligodendrocytes in adult brain (Lim and Alvarez-Buylla, 2016). The olfactory bulb connects entorhinal cortex and influences working memory performance. Adult neurogenesis in the hippocampus is closely related to spatial cognition (Alonso et al., 2024). We then examined whether deletion of Ago2 in NSCs affected cognitive function. Using a novel object recognition assay, we found that WT mice spent a lot of time to explore a novel bead. However, compared to WT mice, cKO mice significantly reduced their responses to a new bead (**Figure 4A** and **B**), indicating that Ago2 knockout in NSCs significantly impaired the animals’ ability to discriminate the novel from the familiar object. Using the MWM assay that detects hippocampal-related spatial learning and memory, we found that Ago2 cKO mice spent significantly more time to locate the hidden platform in the correct quadrant, as indicated by quantitative escape latency compared to WT mice (**Figure 4C** and **D**). In contrast, Ago2 cKO mice spent much less time in the correct quadrant (**Figure 4C** and **D**). These data indicate that Ago2 knockout in NSCs impairs learning and memory.

### Deletion of Argonaute 2 in neural stem cells worsens post-stroke sensorimotor function

**Figure 5:**
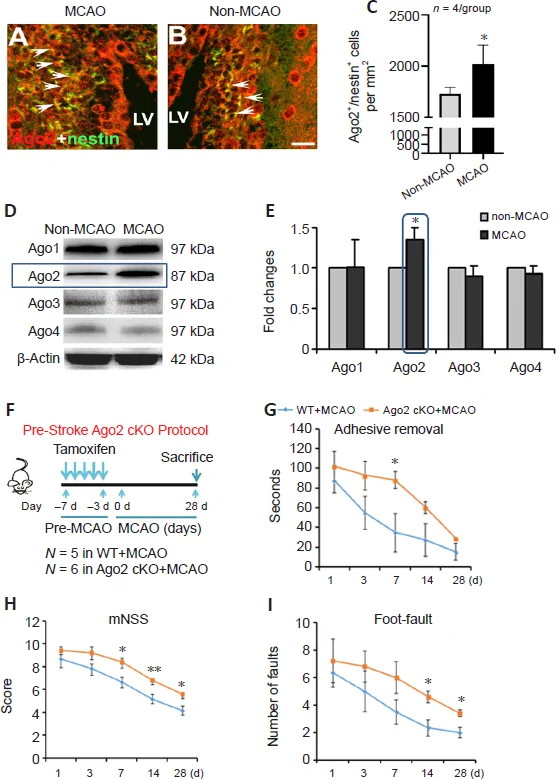
Expression of Ago2 in the V-SVZ after stroke and effect of Ago2 cKO in Ascl1 lineage neural stem cells on neurological outcomes. (A, B) Immunostaining of coronal brain sections shows Ago2^+^/nestin^+^ cells in the ischemic and non-ischemic brains. Scale bar: 100 μm. (C) Quantitative data of Ago2^+^/nestin^+^ cells in the V-SVZ. *n* = 4/group. (D, E) Western blot analysis of V-SVZ neural stem cells harvested from 7 days post-MCAO shows Ago protein levels. (F) Experimental design employed for conditional deletion of Ago2 and when MCAO was performed and when the animals were sacrificed after stroke. (G–I) Conditional ablation of Ago2 in neural stem cells impedes recovery of post-stroke neurological function at 7, 14, or 28 days post-MCAO. Graphs showing outcomes from adhesive removal test (G), mNSS (H), and foot-fault test (I). Significant differences were detected in mNSS, adhesive test from day 7 to 28 post-MCAO between Ago2 cKO and WT mice. *n* = 5–6/group. Data are presented as mean ± SEM. *P < 0.05, **P < 0.01. Ago: Argonaute; cKO: conditional knockout; LV: lateral ventricle; MCAO: middle cerebral artery occlusion; mNSS: modified neurological severity score; V-SVZ: ventricular–subventricular zone; WT: wild-type.

Endogenous neurogenesis in the V-SVZ neurogenic region is correlated with sensorimotor recovery after stroke (Ceanga et al., 2021; Zhao et al., 2024a). To examine whether stroke alters Ago proteins in NSCs, we measured Ago protein levels in the V-SVZ. Immunohistochemistry analysis showed that compared to the non-stroke V-SVZ, stroke significantly increased the coexpression of Ago2^+^ with nestin^+^ cells, a marker of NSCs (Tong and Yin, 2024), in the V-SVZ (**Figure 5A–C**). Western blot analysis of V-SVZ tissues showed that stroke substantially elevated Ago2, but not Ago1, 3, and 4 proteins (**Figure 5D** and **E**). These data suggest that stroke increases Ago2 expression in NSCs. Therefore, we examined if ablation of Ago2 affected the neurological outcomes after stroke (**Figure 5F**). An array of functional assays was conducted. Our data showed that Ago2 cKO mice subjected to MCAO required increased time to remove the adhesive tabs at 7 days post-MCAO (**Figure 5G**), exhibited a significantly reduced score in the mNSS at 7–28 days post-MCAO (**Figure 5H**), and had increased numbers of foot-faults at 14 and 28 days post-MCAO (**Figure 5I**), compared to WT mice after stroke, suggesting the deletion of Ago2 in Ascl1 lineage NSCs and OPCs impaired the neurorecovery after stroke.

**Figure 6:**
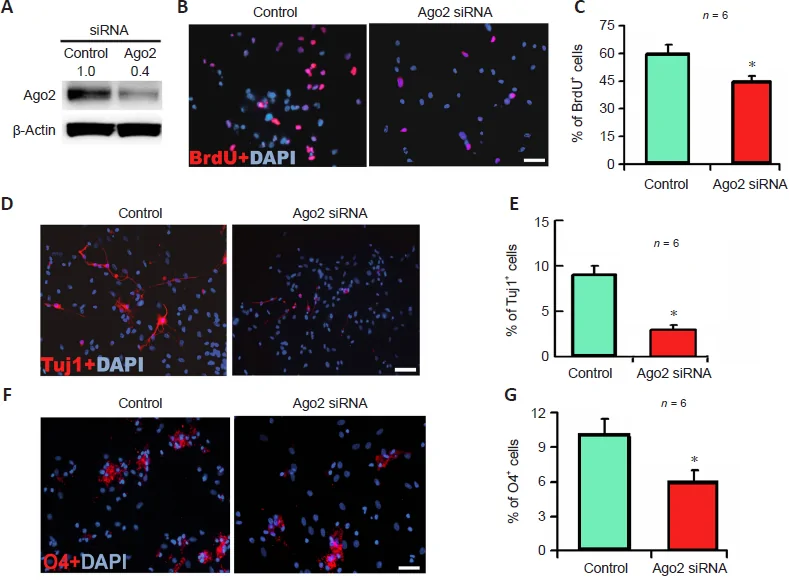
Ablation of Ago2 in ischemic NSCs reduces neurogenesis. (A) Western blot shows that protein levels of Ago2 in NSC cells transfected by siRNA against Ago2 (Ago2) or control siRNA (control). (B, D, F) Immunocytochemistry shows BrdU^+^ (B), Tuj1^+^ (D), and O4^+^ (F) cells in ventricular–subventricular zone NSCs after transfection with siRNA-Ago2 or control siRNA. Scale bars: 20 μm. (C, E, G) Quantitative data analysis shows the percentage of BrdU^+^ (C), Tuj1^+^ (E), and O4^+^ (G) from NSCs. Data are presented as mean ± SEM. *P < 0.05, *vs.* control. Ago2: Argonaute 2; BrdU: bromodeoxyuridine; DAPI: 4′,6′-diamidino-2-phenylindole; NSCs: neural stem cells; siRNA: small interfering RNA; Tuj1: neuron-specific class III β-tubulin.

*In vitro*, we isolated NSCs from the V-SVZ and attenuated endogenous Ago2 in NSCs isolated from ischemic V-SVZ with siRNA specifically against Ago2, which was confirmed with Western blot, and compared these cells with NSCs transfected with scrambled siRNAs (**Figure 6A**). Using *in vitro* experiments, we found that the reduction of endogenous Ago2 by siRNA significantly decreased NSC proliferation (the percentage of BrdU^+^ cells) compared with NSCs transfected scrambled siRNAs (**Figure 6B** and **C**). In addition, under differentiation conditions, knockdown of Ago2 significantly reduced the percentage of Tuj1^+^ neuroblasts (**Figure 6D** and **E**) and O4^+^ OPCs (**Figure 6F** and **G**). These data indicate that Ago2 deletion in Ascl1 lineage cells inhibits the differentiation of NSCs into immature neurons and OPCs.

### Argonaute 2 ablation affects cell transcription post-neurogenesis

**Figure 7:**
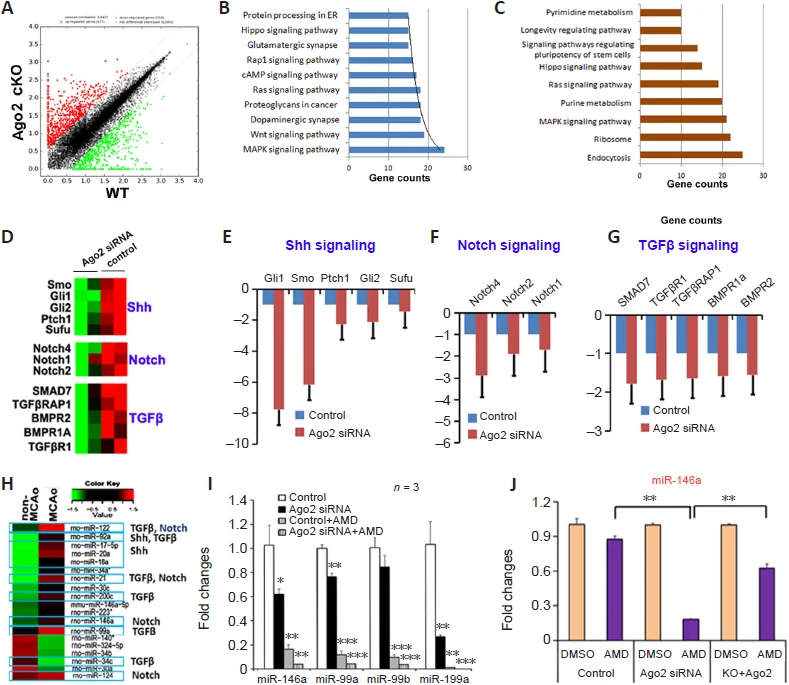
Ago2 ablation in neural stem cells regulates gene transcription and reduces the stability of mature miRNAs. (A) Scatter plots of differentially expressed genes. The values of X and Y axes in the Scatter-Plot are the averaged FPKM (fragments per kilobase per million mapped fragments) values of each group (log2 scaled). Genes above the top line (red dots, up-regulation) or below the bottom line (green dots, down-regulation) indicate more than 1.5 fold change between the two compared groups. Gray dots indicate non-differentially expressed genes. (B, C) Gene ontology (GO) analysis of downregulated (B) and upregulated (C) mRNAs in V-SVZ cells isolated from Ago2 cKO and WT mice using RNA sequencing. *n* = 1/group from pooled RNAs of 3 mice. (D–G) The representative PCR array heatmap (D) and fold changes of genes related to Shh (E), Notch (F) and TGFβ (G) signaling in NPCs with Ago2 ablation with siRNA. The scramble siRNA was employed as a negative control. *n* = 2/group. (H) Deep-sequencing analysis shows a representative partial heatmap of Ago2-CLIP related to Shh, TGFβ Notch signaling pathways in non-ischemic and ischemic V-SVZ tissues. (I) Highly enriched miRNAs in Ago2-RISC are degraded in neural stem cells lacking Ago2 and are further degraded 24 hours after transcription inhibition (AMD). All data were compared with those from the control group (con). *n* = 3/group. (J) Introduction of vector carrying Ago2 cDNA (KO+Ago2) partially but significantly increased miR-146a after transcription inhibition. The data were normalized to the DMSO group. Data are presented as mean ± SEM. *P < 0.05, **P < 0.01, ***P < 0.001, *vs*. control (I). **P < 0.01 (J). Ago2: Argonaute 2; AMD: Actinomycin D; cKO: conditional knockout; siRNA: small interfering RNA; TGFβ: transforming growth factor beta; V-SVZ: ventricular-subventricular zone; WT: wild-type.

To identify the mechanism underlying the effect of Ago2-deficiency on NSCs, we performed a transcriptional analysis of V-SVZ cells isolated from Ago2 cKO and WT mice using RNA-seq at 14 days after initial tamoxifen injection (**Figure 7A**). We found that gene ontology analysis of differentially regulated genes indicated that the functions of downregulated genes were pertinent to the MAPK signaling pathway, Wnt signaling pathway, Dopaminergic synapse, and the Hippo signaling pathway (**Figure 7B**), while those of upregulated genes could be classified into categories such as endocytosis, MAPK signaling pathway, and signaling pathway regulating pluripotency of stem cells (**Figure 7C**).

Furthermore, we identified genes specifically related to adult neurogenesis in Ago2 siRNA-transfected NSCs using a PCR array specifically related to the stem cell-related signaling pathway (Qiagen). We found that Ago2 knockdown in cultured NSCs markedly downregulated multiple genes that mediate the Shh (Gli1, Smo, Ptch1, and Gli2), Notch (Notch1, 2, and 4), and TGFβ (SMAD7, TGFβ1, and BMPR2) signaling pathways (**Figure 7D–G**), which were previously demonstrated to regulate biological functions of NSCs under non-ischemic and ischemic conditions (Liu et al., 2007).

### Argonaute 2 regulates the microRNAs related to neurogenesis after stroke

Ago2 protein is a key player in RISC to affect miRNA processing and function. In our previous study, using a high-throughput Ago2-based RNA immunoprecipitation to immunoprecipitated Ago2-RNA complexes followed by miRNA sequencing (Ago2 RIP-seq) (Zhao et al., 2024b), we discovered that stroke substantially altered the profiles of Ago2-bound miRNAs in cultured NSCs harvested from non-ischemic and ischemic V-SVZ of animals subjected to 7 days of MCAO (**Figure 7H**). Among these miRNAs, we found that a number of miRNAs were previously experimentally validated to be related to genes that are involved in the signaling pathways of Shh (miR-17a, miR-18a, miR-20a, and miR-92a, and members of miR17-92 cluster) (Uziel et al., 2009; Liu et al., 2013), Notch (miR-124, miR-34c, miR-146a, miR-21, and miR-122) (Wang et al., 2010; Zoni et al., 2015), and TGFβ (miR-21, miR-122, miR-200c, miR-92a, miR-30a, and miR-99) (Zoni et al., 2015; **Figure 7H**). These signaling pathways are known to modulate the renewal and differentiation of NSCs, which were consistent with our RNA-seq data showing the alteration of signaling pathway regulating pluripotency of stem cells (**Figure 7D**).

Mechanisms underlying Ago2-regulated miRNA function are unknown in adult NSCs. Our data showed that knockdown of Ago2 in cultured NSCs significantly reduced the abundance of stroke-increased mature miRNAs, including miR-146a and miR-99a (**Figure 7I**). A recent study indicates that Ago2 post-transcriptionally regulates the stability of mature miRNAs (Winter and Diederichs, 2011). We then tested whether Ago2 ablation affected miRNA abundance via regulating the mature miRNA stability in NSCs. MiRNA decay was investigated after treatment with Actinomycin D (AMD), a transcription inhibitor, which prevents on-going transcription. Our data showed that treatment of NSCs transfected by siRNA against Ago2 with AMD for 24 hours led to a substantial degradation of stroke-increased miR-146a, miR-99a, and others in Ago2-ablated NSCs compared to levels in NSCs transfected by scramble siRNA (**Figure 7I**). In contrast, upregulation of Ago2 by transfection of vector carrying Ago2 cDNA into NPCs partially rescued miR-146a degraded by AMD (**Figure 7J**). These data suggest that the reduction of mature miRNAs by ablation of Ago2 is induced by the loss of miRNA stability.

## Discussion

In this study, using Ago2-cKO mice we investigated the functional roles of NSC-specific Ago2 in adult neurogenesis, oligodendrogenesis, and neurological function. Our data demonstrate that specific deletion of Ago2 in Ascl1 lineage NSCs substantially reduces the proliferation and differentiation of NSCs into neuroblasts and OPCs, leading to impairments of learning and memory function. Knockdown of Ago2-cKO also impairs neurological recovery in ischemic mice. Furthermore, our data reveal an important regulatory mechanism of NSC Ago2 in mediating Ago2-mRNA interactions via the regulation of enriched miRNA stability. These data demonstrate that Ago2 in adult NSCs is required for adult neurogenesis and oligodendrogenesis under non-ischemic and ischemic conditions.

Our data demonstrate that adult NSCs, neuroblasts, and OPCs express Ago2. Ascl1 is expressed in progenitors of both neurons and oligodendrocytes, but not astrocytes (Zhang et al., 2011). Ascl1 lineage NSCs and OPCs contribute to stroke-induced neurogenesis and oligodendrogenesis (Zhang et al., 2011). Therefore, a conditional Ago2 transgenic mouse line in Ascl1 lineage cells was employed. Neurogenesis impacts cognitive function (Alonso et al., 2024; Geigenmüller et al., 2024; Ammothumkandy et al., 2025). Oligodendrocytes produce the myelin sheath and provide support to axons, which are highly correlated with cognition. In addition to OPCs in the parenchyma, NSCs in the V-SVZ are an important source of oligodendrocytes in the adult brain during different stages of development that involve the generation of OPCs, which can differentiate into mature oligodendrocytes and express myelin proteins (Franklin and Ffrench-Constant, 2008; Zhang et al., 2011). Adult oligodendrogenesis facilitates myelination. Damage to oligodendrocytes contributes to dementia. Our data suggest that Ago2 ablation in Ascl1 lineage cells influences oligodendrogenesis in both V-SVZ neurogenic and CC areas. Although our data demonstrated that loss of Ago2 reduced the differentiation and maturation of OPCs, we do not exclude the possibility that Ago2 deletion may affect the survival of OPCs. Our data suggest that the reduction of neurogenesis and oligodendrogenesis by deletion of Ago2 is closely associated with the impairment of post-stroke functional recovery.

Since Ago2 was conditionally knocked out only in the Ascl1 lineage cells of transgenic mice instead of all cell types in the adult brain, its deletion did not lead to the dysfunction of all miRNAs, but only affected Ago2-enriched miRNA and mRNA networks associated with neurogenesis and oligodendrogenesis. Furthermore, our previous study demonstrated that Ago2-immunoprecipitation followed by high-throughput sequencing (Ago2 RIP-seq) revealed that stroke substantially altered the profile of Ago2-bound miRNAs, including miR-146a, when compared to non-stroke NSCs (Liu et al., 2017b). MiR-146 regulates stroke-induced neurogenesis and oligodendrogenesis (Liu et al., 2017a). Reduction of Ago2 in cultured NSCs using siRNA against Ago2 inhibited these miRNAs and significantly decreased stroke-induced NSC proliferation, and neuronal and oligodendrocyte differentiation. These data suggest that Ago2 plays an important role in regulating stroke-induced neurogenesis and oligodendrogenesis by mediating miRNAs in NSCs via post-transcriptional mechanism.

Recent work has uncovered the critical role of the Ago protein that controls the degradation of mature miRNAs (Winter and Diederichs, 2011). Using a miRNA decay assay, we found that miRNAs are stable in NSCs from WT mice, however, Ago2-enriched miRNAs degrade rapidly in NSCs lacking Ago2. This suggests that loss of endogenous Ago2 loading destabilizes mature miRNAs, promotes their degradation, and gives rise to decreased half-lives of miRNAs. Although studies have focused on miR-146a, we are aware that Ago2 can maintain the stability of many mature miRNAs in NSCs. However, it is noted that not all miRNAs were reduced in Ago2-deleted cells. Although previous studies have suggested that the dysregulation of Ago2 influences various biological processes including angiogenesis, cancer, and adipogenesis by perturbing the expression of specific miRNAs, our study revealed that post-stroke dysregulation of Ago2 induces discernible alterations in AGO2-controlled miRNAs within NSCs (Asai et al., 2008; Ye et al., 2015). These findings strongly imply that Ago2 modulates a distinct subset of miRNAs in a manner dependent on cell type, particularly in response to ischemic stroke. Given the role of Ago2 in miRNA stability, increased activity of the Ago2 protein may improve the efficacy of miRNAs in the treatment of neurological disorders.

This study has some limitations that should be noted. In addition to its role in transcriptional gene silencing mediated by miRNAs, Ago2 has been observed to localize within the nucleus, where it participates in chromatin remodeling (Xie et al., 2024) and in alternative RNA splicing through recruitment of RNA Pol II or splicing factors (Ameyar-Zazoua et al., 2012; Sharma et al., 2016). These epigenetic factors and alterative RNA splicing play pivotal roles in neurogenesis and oligodendrogenesis (Colussi and Grassi, 2021; Bernou et al., 2024; Kunoh et al., 2024; Mu et al., 2025). Therefore, Ago2 may regulate gene expression at both transcriptional and post-transcriptional levels. Further investigation of the role of Ago2 in transcriptional regulation may provide new insight into mechanisms underlying neurorecovery following stroke.

In conclusion, our data provide novel evidence that Ago2 plays an important role in stroke-induced neurogenesis and oligodendrogenesis by regulating the steady-state expression levels of mature miRNAs. This finding highlights Ago2 as a potential therapeutic target for enhancing miRNA function in the ischemic brain.

## Data Availability

*No additional data are available.*
